# Characteristics, predictors, and management of post-extubation dysphagia in critically Ill children: a scoping review

**DOI:** 10.3389/fped.2026.1854127

**Published:** 2026-06-15

**Authors:** Yaokang Xue, Baorong Zhang, Xiaoshuang Zhao, Lili Hu, Liting Ju, Hairui Sun, Dan Zhang, Jiping Zhang, Yuxuan Yang, Ruixin Guan

**Affiliations:** 1 Department of Pediatric Intensive Care Unit, The First Hospital of Jilin University, Changchun, China; 2 School of Nursing, Jilin University, Changchun, China; 3 Department of Nursing, The First Hospital of Jilin University, Changchun, China

**Keywords:** management strategies, pediatric intensive care, pediatrics, post-extubation dysphagia, predictors, scoping review

## Abstract

**Background:**

Post-extubation dysphagia (PED) is a common complication in critically ill children, associated with aspiration, malnutrition, and prolonged hospitalization. Standardized pediatric-specific assessment and management pathways are lacking, and published studies show substantial heterogeneity in diagnostic criteria, assessment timing, and intervention strategies.

**Objective:**

This scoping review maps available evidence on pediatric PED, addressing: (1) incidence and clinical characteristics; (2) categories and effect sizes of independent predictors; and (3) the current state of prediction tools, bedside screening instruments, and management strategies.

**Methods:**

Nine databases were searched systematically—PubMed, Embase, Cochrane Library, Web of Science, CINAHL, CNKI, Wanfang, VIP, and CBM—with a search cutoff of 12 March 2026. Fifteen studies were included, encompassing cohort studies, randomized controlled trials, quasi-experimental studies, and scale-development studies. Data were synthesized narratively following the JBI scoping review methodological framework.

**Results:**

Included studies were published between 2018 and 2026; 73.3% (11/15) appeared after 2022, originating primarily from mainland China (*n* = 10) and Brazil (*n* = 3). The clinical apparent incidence ranged from 28.36% to 68.94%. No included bedside assessment instrument was validated against VFSS or FEES,representing a central methodological gap in the current evidence base.Independent predictors were categorized into three evidence tiers: Tier 1: intubation duration, age, and IWS; Tier 2: neurological comorbidities and number of intubations ≥2; Tier 3:delirium.The CRISPED score is the only prediction tool with temporal internal validation (C-statistic 0.85–0.86). No assessment instrument has been validated against an instrumental gold standard. Interventions centered on oral motor training, with no standardization in frequency, intensity, or multidisciplinary integration.

**Conclusion:**

Pediatric PED incidence is high and core risk factors have been repeatedly reported across multiple multivariable models, but the field shows substantial heterogeneity in diagnostic definitions, assessment tools, and intervention protocols. The absence of instrumental validation for bedside assessment tools is the central limitation. Key gaps include the absence of gold-standard diagnostic accuracy studies, externally validated prediction models, and standardized intervention pathways. Future research should prioritize multicenter prospective studies to unify diagnostic criteria, develop age-appropriate bedside screening tools, and establish a nurse-led multidisciplinary management framework.

**Systematic review registration:**

Open Science Framework (OSF), identifier doi: 10.17605/OSF.IO/XSJ9B.

## 1 Introduction

Endotracheal intubation with mechanical ventilation is one of the most critical life-support interventions in the PICU; approximately 35%–40% of PICU patients require invasive mechanical ventilation (1, 2). Mechanical injury to the laryngopharynx caused by intubation, local mucosal inflammation, and altered pharyngeal muscle function can disrupt normal swallowing coordination and give rise to post-extubation dysphagia (PED) (3, 4). Clinical manifestations include choking, swallowing difficulty, food spillage, and voice change. Related adverse outcomes encompass aspiration pneumonia, malnutrition, unplanned re-intubation, and prolonged hospitalization (4, 5), and PED is associated with poor short-term outcomes and elevated risk of long-term developmental impairment.

Adult critical care research has established a clear disease burden for PED and demonstrated its independent association with adverse clinical outcomes (6). Instrumental assessment studies suggest that bedside clinical observation may underestimate its true prevalence (7). By contrast, pediatric PED research is more recent. Reported incidence ranges from 28.36% to 68.94% (5, 8–13), with substantial methodological heterogeneity. Identified risk factors span young age, prolonged intubation, repeated intubation, neurological comorbidities, IWS, and delirium (5, 8–12). Available assessment tools are largely adapted from adult instruments and lack pediatric-specific validation (11, 13–20). Oral motor training interventions have shown preliminary efficacy, but studies differ markedly in assessment timing, tool selection, and intervention protocols (14–20). The pediatric airway and laryngopharynx are in continuous developmental flux, and the underlying neuromuscular control of swallowing differs from that in adults (21, 22), making direct translation of adult PED assessment tools and clinical prediction models unreliable. Although pediatric PED research has grown, the literature remains scattered and methodologically inconsistent, with no established consensus on assessment timing, instrument selection, or intervention pathways. Systematically synthesizing pediatric-specific evidence is a necessary foundation for building a clinical pathway for post-extubation swallowing management in the PICU.

This scoping review follows Arksey and O'Malley's scoping review framework as updated by JBI guidelines, with reporting according to the PRISMA-ScR checklist (23, 24), and addresses four core questions: (1) the incidence and main contributing factors for PED in critically ill children; (2) the performance and validation status of available clinical prediction tools; (3) the standardization of PED screening timing and bedside assessment instruments; and (4) the interventions for pediatric PED, including implementation personnel, initiation timing, specific methods, and outcome measures. Building on the evidence synthesis, this paper proposes the Nurse-led Risk-stratified Management Framework for Post-extubation Dysphagia (NRMPF-PED) as a hypothetical conceptual model for future research, to provide a structured theoretical reference for future implementation research and PICU management optimization.

## 2 Methods

### 2.1 Methodological framework and reporting standards

This study used Arksey and O'Malley's five-stage scoping review framework (25) as the foundational methodological structure. JBI updated methodological guidelines (23) informed the refinement of the PCC framework, data extraction procedures, and narrative synthesis approach. Reporting followed the PRISMA-ScR checklist (24). The study was prospectively registered on the Open Science Framework (OSF; DOI: 10.17605/OSF.IO/XSJ9B). Research questions were structured using the PCC (Population, Concept, Context) framework. Population: critically ill children aged ≤18 years who underwent endotracheal intubation with mechanical ventilation and were successfully extubated in the PICU. Concept: incidence and clinical characteristics of PED, independent risk factors and clinical prediction tools, bedside screening and instrumental assessment methods, and related interventions and outcomes. Context: the PICU clinical setting, including single-center and multicenter studies from institutions with varying resource levels.

### 2.2 Core research questions

Four core questions guided the review: (1) the incidence and main contributing factors for PED in critically ill children; (2) the performance and validation status of available clinical prediction tools; (3) the standardization of PED screening timing and bedside assessment tools; and (4) the initiation timing, implementing personnel, specific methods, and outcome indicators for PED interventions.

### 2.3 Eligibility criteria

Inclusion criteria: (i) critically ill children aged ≤18 years following endotracheal intubation and extubation; (ii) studies addressing PED incidence and risk factors, clinical prediction scores or models, swallowing screening and assessment tools, or rehabilitation interventions for pediatric PED with reported outcomes; (iii) any primary study design, including randomized controlled trials, observational studies, quasi-experimental studies, scale-development studies, and case series;(iv) full text available; (v) language restricted to Chinese or English. No lower date restriction was applied; the earliest study meeting all PCC criteria was da Silva et al. (26), and no eligible study predating this was identified across 3,509 retrieved records.

Exclusion criteria: (i) dysphagia unrelated to endotracheal intubation; (ii) patients with tracheotomy; (iii) mixed adult–pediatric populations that did not report pediatric data separately; (iv) full text unavailable; (v) conference abstracts, editorial comments, and duplicate publications; (vi) studies that did not clearly distinguish post-extubation dysphagia from dysphagia attributable to other causes.(vii) narrative review articles and secondary literature syntheses.

### 2.4 Search strategy

Nine databases were searched systematically: PubMed, Embase, Cochrane Library, Web of Science, CINAHL, CNKI, Wanfang, VIP Chinese Journal Service Platform, and the Chinese Biomedical Literature Database (CBM). The final search date was 12 March 2026. The search strategy combined controlled vocabulary with free-text terms across three conceptual dimensions. English terms included: Child, Child Preschool, Adolescent, Pediatrics, Airway Extubation, respiration artificial, extubation, postextubation, post-extubation, and Deglutition Disorders. The core PubMed strategy: (“Intubation, Intratracheal"[Mesh] OR “intubation"[tiab] OR “endotracheal tube"[tiab] OR “mechanical ventilation"[tiab] OR “extubation"[tiab]) AND (“Deglutition Disorders"[Mesh] OR “dysphagia"[tiab] OR “swallowing disorder"[tiab] OR “swallowing dysfunction"[tiab]) AND (“Child"[Mesh] OR “Infant"[Mesh] OR “pediatric"[tiab] OR “paediatric"[tiab] OR “child"[tiab] OR “infant"[tiab] OR “neonat"[tiab] OR “toddler"[tiab]). Reference lists of included studies were hand-searched. The full search strategy is provided in Supplementary File S1.

### 2.5 Study selection and data extraction

Duplicates were removed using EndNote 21.0. Two reviewers independently screened titles and abstracts, followed by full-text review; inter-rater agreement was *κ* = 0.87. Disagreements were resolved by discussion; unresolved cases were adjudicated by a third reviewer. Standardized extraction forms captured: basic study information (first author, year, country, study design), participant characteristics (sample size, age, PICU type), PED incidence, risk factors and effect sizes (OR and 95% CI), assessment tool type and timing, prediction model performance metrics (C-statistic, AUC, sensitivity, specificity), specific intervention technique combinations, and outcome measures.

### 2.6 Data synthesis

Given substantial heterogeneity across included studies in methodological design, assessment instruments, and outcome measures, data were synthesized narratively without quantitative pooling. Consistent with Arksey and O'Malley's scoping review framework as updated by JBI guidelines (23), no formal risk-of-bias assessment was performed and no GRADE certainty ratings were assigned. Study design type and known methodological limitations are described objectively throughout the narrative synthesis.

## 3 Results

### 3.1 Search results and study selection

**Figure 1:**
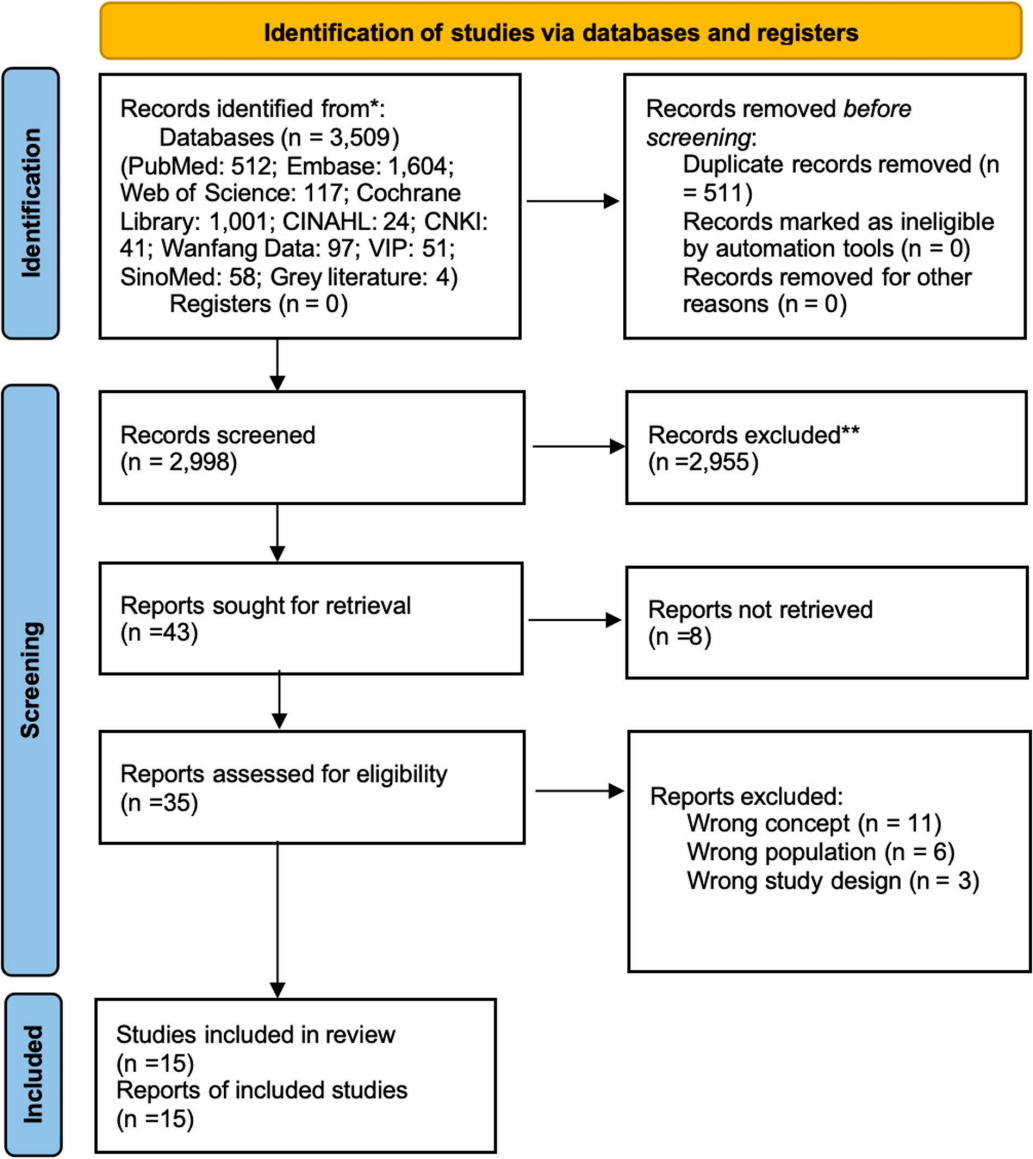
PRISMA-ScR flow diagram of study selection. A total of 3,509 records were retrieved from 9 databases. After deduplication (*n* = 2,998), title and abstract screening (*n* = 43 retrieved), and full-text review, 15 studies were included in the final synthesis.

Database searches retrieved 3,509 records. After deduplication, 2,998 remained. Title and abstract screening excluded 2,955 records, leaving 43 for full-text review. Full-text review excluded 28 records: full text unavailable (*n* = 8), age of study population outside the inclusion criteria (*n* = 6), dysphagia not related to endotracheal intubation (*n* = 11), and ineligible study design (*n* = 3). The three records excluded on this basis were narrative review articles, consistent with exclusion criterion (vii) in Section 2.3. Fifteen studies were included. Inter-rater agreement was *κ* = 0.87. The study selection flow is presented in Figure 1 (PRISMA-ScR flow diagram).

### 3.2 Characteristics of included studies

The 15 studies were published between 2018 and 2026; no lower date restriction was applied in our search strategy, and this range reflects the natural distribution of eligible primary studies (no eligible primary study meeting all PCC criteria was identified before 2018). 73.3% (11/15) appeared after 2022. Study designs included prospective cohort studies (*n* = 2),5,10 retrospective studies (*n* = 4),8,9,12,14 a cross-sectional scale-development and validation study (*n* = 1),13 randomized controlled trials (*n* = 3),15,16,20 and quasi-experimental studies (*n* = 5).17,18,27 Sample sizes ranged from 53 to 432 participants; patient age ranged from 0 to 16 years. All 13 journal articles were published in peer-reviewed journals. The remaining two studies are master's theses (11, 13); in China's academic degree system, master's theses undergo external blind peer review and a public oral defense before institutional registration in CNKI, where both are publicly accessible. Their findings should nonetheless be interpreted with appropriate caution relative to journal-published data. The da Silva team contributed the most studies (*n* = 3), covering intervention research (26), risk factor analysis (5), and prediction model development (10). Characteristics of all included studies are presented in Table 1.

**Table 1:** Basic characteristics of the included studies (for oversized tables or figures, the font size may be reduced to 7.5pt.).

No	Author (Year) Country	Publication type	Study design	Sample size (n)	Age (age group)	Primary diagnosis/patient population	PED incidence	Assessment tool (assessor)	Assessment timing	Key risk factors reported	Research focus
1	da Silva (10) (2026)Brazil	Journal Article	Prospective cohort	432 (Dev:300; Val:132)	1 mo – 15 yr (infants, children, adolescents)	Critically ill; majority respiratory	Overall 65.04%	P-FOIS (SLP)	24–48 h post-extubation	IWS; number of intubations; intubation duration	Incidence, risk factors, prediction model
2	Zhu (9) (2025)China	Journal Article	Retrospective observational	84 (PED 40)	≤3 yr vs. >3 yr (toddlers vs. children)	MV children (respiratory, neurological, metabolic)	47.62%	SSA (Nurses)	Within 24 h	Age ≤3 yr; neurological impairment; intubation ≥72 h; gastric tube ≥72 h; IWS	Incidence, risk factors, prediction model
3	Hu L (11) (2025)China	Master's Thesis	Prospective observational	216 (PED 121)	3–14 yr (children, adolescents)	Respiratory 65.74%; neurological 11.11%	56.02%	P-GUSS-ICU (Nurses)	4 h	Age; delirium; intubation duration; intubations ≥2 times	Incidence, risk factors, scale development
4	Chen (16) (2025)China	Journal Article	RCT	112 (IG 56, CG 56)	36–41 wk GA (neonates)	PICU infants with acquired dysphagia	N/A	NOMAS (NR)	On regaining consciousness	N/A	Intervention
5	Mo (14) (2024) China	Journal Article	Retrospective comparative	120(IG 60, CG 60)	6–24 mo (infants, toddlers)	Severe pneumonia with post-extubation dysphagia	N/A	SSA, FIDS (NR)	4 h,24 h,48 h,72 h, discharge	N/A	Intervention
6	Yogo (8) (2024) Japan	Journal Article	Retrospective case-series	53 (PED 22)	median 58 mo (children)	Pediatric trauma (83.00% TBI)	41.51%	MWST, trial feeding (Nurses)	Within 72 h	High head/neck AIS; longer intubation; emergent intubation; barbiturate	Incidence, scale development
7	Wang (13) (2024) China	Master's Thesis	Cross-sectional (scale development)	134 (validation phase)	2–14 yr (toddlers, children, adolescents)	PICU post-extubation after MV	28.36%	PED scale (PED-C self-developed) (Nurses vs. SLP/MD)	2–24 h	N/A	Incidence, risk factors, scale validation
8	Li (18) (2023) China	Journal Article	Quasi-experimental	100(IG 50, CG 50)	1–3 yr (toddlers)	Acquired dysphagia in PICU	N/A	SSA, DOSS (NR)	On clinical stabilization	N/A	Intervention
9	Zhou (17) (2023) China	Journal Article	Quasi-experimental (historical control)	95(IG 39, CG 56)	1–3 yr (toddlers)	Acquired dysphagia (severe pneumonia, encephalitis, HFMD)	N/A	NOMAS, FLACC, SGNA (multidisciplinary team)	When meeting early rehab indications	N/A	Intervention
10	da Silva (5) (2023) Brazil	Journal Article	Prospective cohort	161 (PED 111)	1 mo – 15 yr (median 5 mo) (infants, children, adolescents)	Critically ill, MV >24 h	68.94%	FOIS (SLP)	24–48 h	Age <24 mo; neurological comorbidities; IWS; NMBA; intubation >72 h; cuffed ETT (protective)	Incidence, risk factors
11	Liu L (19) (2022) China	Journal Article	Quasi-experimental (historical control)	100(IG 50, CG 50)	1–3 yr (toddlers)	Acquired dysphagia after intubation	N/A	SSA, MWST (NR)	Day of extubation	N/A	Intervention
12	Liu Q (20) (2022) China	Journal Article	RCT	60 (IG 30, CG 30)	1 mo – 2 yr (infants, toddlers)	Acquired dysphagia after intubation	N/A	NOMAS (NR)	On vital sign stabilization	N/A	Intervention
13	Hoffmeister (12) (2019) USA	Journal Article	Retrospective cohort	372 (PED 108)	0–16 yr (median 29 mo) (neonates, infants, toddlers, children, adolescents)	ICU extubated, oral feeding attempt within 72 h	29.03%	Clinical flowsheet or SLP-CSE (Nurses/SLP/MD)	First feeding (within 72 h)	Age 0–24 mo; trauma diagnosis; intubation duration (per hour)	Incidence, risk factors
14	Zhang (15) (2019) China	Journal Article	RCT	80 (IG 40, CG 40)	1–24 mo (mean 7.6 mo) (infants, toddlers)	Post-extubation after MV	N/A	SSB (Nurses)	>24 h, conscious, stable	N/A	Intervention
15	da Silva (26) (2018) Brazil	Journal Article	Quasi-experimental (historical control)	115(HG 41, IG 74)	1 mo – 15 yr (median 3 mo) (infants, children, adolescents)	Intubated >24 h, FOIS ≤3	N/A	FOIS, BSE (SLP)	Within 24 h of extubation and at discharge	N/A	Intervention

PED, post-extubation dysphagia; IG, intervention group; CG, control group; HG, historical control group; Dev, derivation cohort; Val, validation cohort. mo, months; yr, years; wk, weeks. SLP, speech-language pathologist; MD, physician. P-FOIS, Pediatric Functional Oral Intake Scale; SSA, Standardized Swallowing Assessment; P-GUSS-ICU, Pediatric Gugging Swallowing Screen-ICU; NOMAS, Neonatal Oral-Motor Assessment Scale; FIDS, Feeding Interaction and Development Scale; MWST, Modified Water Swallowing Test; TF, trial feeding; PED-C, Post-extubation Dysphagia in Children screening tool; DOSS, Dysphagia Outcome and Severity Scale; CF, clinical feeding evaluation; SLP-CSE, SLP clinical swallow evaluation; BSE, bedside swallowing evaluation; SSB, suck–swallow–breathe coordination scale; NR, not reported.N/A, not applicable.

### 3.3 incidence and risk factors

#### 3.3.1 Incidence

Seven studies reported PED incidence, with estimates ranging from 28.36% to 68.94%: da Silva et al. (5) reported 68.94%; Hu (11) 56.02%; Zhu et al. (9) 47.62%; Yogo et al. (8) 41.51%; Hoffmeister et al. (12) 29.03%; Wang (13) 28.36%; and da Silva et al. (10) an overall rate of 65.0% (derivation cohort 68.3%, validation cohort 57.6%). Variation across studies reflects differences in assessment timing, assessment tools, and patient case mix (Table 1).

#### 3.3.2 Independent risk factors

Six studies reported independent risk factors (5, 8–12) (Table 2).Based on available evidence, these predictors can be classified into three tiers. The first tier comprises factors reported across multiple independent studies: intubation duration was reported in five studies, age in four studies, and iatrogenic withdrawal syndrome (IWS) in three studies. The second tier includes emerging predictors requiring further validation: neurological comorbidities and number of intubations(≥2 times),each reported in two studies. The third tier includes a hypothesis-generating single-center finding: delirium was reported in only one single-center study and requires further validation. Additionally, Yogo et al. (8) reported associations of severe head trauma and longer ventilator support time with PED in bivariate analysis (*P* < 0.05), but did not perform multivariable regression. da Silva et al. (5) identified the use of a cuffed endotracheal tube as a protective factor.

**Table 2:** Effect sizes of independent risk factors for pediatric post-extubation dysphagia (for oversized tables or figures, the font size may be reduced to 9pt.).

Author (Year) Country	Patient clinical characteristics (setting)	Key outcome measure (timing)	Independent risk factors (definition/cut-off)	OR/aOR(95%CI)	*P*-value
da Silva (2026) Brazil	*n* = 432,Mixed medical-surgical PICU	P-FOIS by SLP,Within 24–48 h post-extubation	IWS(Diagnosed via SOS scale≥4)	8.27 (4.13–16.50)	<0.001
			Number of intubations(per episode)	1.70 (1.14–2.54)	0.01
			Intubation duration(per day)	1.28 (1.16–1.40)	<0.001
Zhu (2025) China	*n* = 84, Mixed PICU patients	SSA by Nurses,Within 24 h post-extubation	Age(≤3 yr vs. > 3 yr)	4.789 (1.576–11.232)	<0.01
			Neurological impairment(Presence of CNS disease)	6.234 (2.778–13.632)	<0.01
			Midazolam infusion(Continuous intravenous)	5.56 (1.076–16.314)	<0.01
			Intubation duration(≥72 h)	4.544 (2.973–15.732)	<0.01
			Gastric tube duration(≥72 h)	7.123 (1.469–9.237)	<0.01
			IWS(Clinical withdrawal scales)	3.454 (1.176–13.652)	<0.01
Hu L (2025) China	*n* = 216, Mixed PICU patients	P-GUSS-ICU by Nurses,At 4 h post-extubation	Age(per yr)	0.908 (0.836–0.987)	0.023
			Delirium(Positive screening)	2.608 (1.211–5.620)	0.014
			Intubation duration(per h)	1.008 (1.001–1.015)	0.033
			Intubations(≥2 times)	3.398 (1.717–6.725)	<0.001
Hoffmeister (2019) USA	*n* = 372, Tertiary multidisciplinary PICU	SLP-CSE,Within 72 h post-extubation	Age(0–24 mo vs. ≥ 25 mo)	2.627 (1.151–5.997)	0.022
			Trauma diagnosis	5.058 (1.567–16.321)	0.007
			Intubation duration(per h)	1.017 (1.012–1.023)	<0.001
da Silva (2023) Brazil	*n* = 161, Mixed medical-surgical PICU	FOIS by SLP,Within 24–48 h post-extubation	Age(<24 mo)	4.84 (1.50–15.60)	0.008
			Neurological comorbidities	7.47 (1.36–40.96)	0.028
			IWS(SOS score≥4)	5.52 (1.31–23.14)	0.019
			NMBA use	4.19 (1.18–14.82)	0.025
			Intubation(>72 h)	3.22 (1.08–9.64)	0.036
			Cuffed ETT(Protective factor)	0.35 (0.13–0.95)	0.039
Yogo (2024) Japan	*n* = 53, Pediatric trauma patients	MWST by Nurses,Within 72 h post-extubation	Severe head trauma(AIS≥4)*	N/A	<0.05
			Longer ventilator support time*	N/A	<0.05

IWS, iatrogenic withdrawal syndrome; NMBA, neuromuscular blocking agent; ETT, endotracheal tube; AIS, Abbreviated Injury Scale; SOS, Sophia Observation Withdrawal Symptoms Scale; OR, odds ratio; aOR, adjusted odds ratio; CI. An OR < 1 (e.g., cuffed ETT) indicates a protective effect.

* indicates that the values reported by Yogo (2024) reports bivariate analysis only (multivariable regression not performed), hence OR is not applicable (N/A). All other OR/aOR values are from multivariable models.

### 3.4 clinical prediction tools

Two studies reported clinical prediction tools for pediatric PED (9, 10) (Table 3). Key performance characteristics are presented in Table 3; the following highlights methodological points not shown there. (1) The CRISPED score (10) incorporates three bedside-obtainable variables (IWS, number of intubations, intubation duration) and is the only pediatric PED prediction tool with temporal internal validation (C-statistics 0.85 and 0.86 for derivation and validation cohorts). Its three-tier risk stratification provides an actionable basis for resource allocation. (2) The logistic regression model by Zhu et al. (9) has apparent validation only (Hosmer–Lemeshow test); the EPV ratio of approximately 6.7 falls below the recommended ≥10 threshold, suggesting potential overfitting, and no external or temporal validation has been performed.

**Table 3:** Characteristics of prediction models for pediatric post-extubation dysphagia.

Study	Model name/type	Core predictors	Performance (AUC)	Validation method
da Silva (10) (2026) Brazil	CRISPED Clinical Risk Score	IWS Number of intubations Duration of intubation	Derivation: 0.85 Validation: 0.86	Temporal internal validation (Split by admission period)
Zhu (9) (2025) China	Logistic Regression Prediction Model	Age ≤ 3 yr Neurological impairment Midazolam infusion Intubatio*n* ≥ 72 h Gastric tube ≥ 72 h IWS	Overall: 0.791 (95% CI: 0.688–0.894)	Apparent validation (Hosmer-Lemeshow test only; no external or temporal validation)

CRISPED: Clinical Risk Score for Post-extubation Dysphagia. IWS: Iatrogenic Withdrawal Syndrome.

### 3.5 assessment tools and implementing personnel

#### 3.5.1 Types of assessment tool

Assessment tools used in the included studies fall into three categories (Table 1). (1) Functional scales: the Pediatric Functional Oral Intake Scale (P-FOIS) was used in three studies (5, 10, 26) and the Standardized Swallowing Assessment (SSA) (27) in four studies (9, 14, 18, 19). (2) Water-swallow tests: the Modified Water Swallowing Test (MWST) (28) was used in two studies (8, 19). (3) Pediatric-specific scales: the PED-C scale developed by Wang (13) applies to children aged 2–14 years, consists of 10 items, and demonstrated Cronbach's *α* = 0.834, inter-rater reliability 0.853, content validity 0.98, AUC 0.910, and—at a cut-off of 5—sensitivity 89.47% and specificity 83.33%, with a median assessment time of 7 min. The P-GUSS-ICU adapted by Hu (11) applies to children aged 3–14 years, uses a two-stage incremental water challenge, and showed content validity 0.96 and *κ* = 0.91. No study used videofluoroscopic swallow study (VFSS) or fiberoptic endoscopic evaluation of swallowing (FEES).

#### 3.5.2 Assessment timing

Ten studies explicitly reported assessment timing (5, 8–15, 26). In observational studies, assessment occurred at 2–24 h post-extubation (13), 4 h (11), within 24 h (9), 24–48 h (5, 10), and within 72 h (8, 12). In interventional studies, timing ranged from 4 h (14) to within 24 h (26) and ≥24 h three times daily (15). Five studies did not explicitly report assessment timing. No study compared the effect of different time points on diagnostic accuracy or clinical outcomes.

#### 3.5.3 Implementing personnel

Ten of 15 studies explicitly reported assessor professional background. Among observational studies, two were conducted by speech-language pathologists (SLPs) (5, 10), four by nurses (8, 9, 11, 13), and one used multidisciplinary collaboration (12). Among interventional studies, one was conducted by SLPs (26) and two by nurses (14, 15). The remaining five interventional studies did not specify assessor professional background (Table 1).

### 3.6 Management models and interventions

#### 3.6.1 Macroscopic management frameworks

Four studies addressed broader management framework development (Table 4). Da Silva et al. (26) established an SLP-led multidisciplinary care pathway; the intervention group showed higher rates of complete oral feeding at discharge (100% vs. 78%) and a lower re-intubation rate. Zhou et al. (17) built a “4F” nursing management model (Full-time, Full-process, Full-system, Full-dimensional), with the intervention group showing improvements in NOMAS (63) scores, length of stay, nasogastric tube (NGT) dwell time, and nutritional status. Chen & Lou (16) applied a goal-directed nursing pathway to PICU infants with acquired dysphagia. Li et al. (18) used a goal-driven staged rehabilitation training program.

**Table 4:** Summary of clinical management frameworks and pathways for pediatric PED.

Author (Year) Country	Management framework / pathway	Core dimensions & mechanisms	Multidisciplinary / team focus
Zhou (17) (2023) China	4F Nursing Management Model	Integrates “Full-time, Full-process, Full-system, Full-dimensional” concepts. Shifts from fragmented interventions to holistic management covering environmental, physio-psychological needs, and continuous caregiver involvement.	Nurse-led(with family caregiver integration)
Chen (16) (2025) China	Goal-Directed Nursing Pathway	Divides the clinical course into predefined stages with quantifiable recovery goals. Reduces arbitrary clinical decisions by enforcing pathway-driven timelines.	Nurse-led
Li (18) (2023) China	Goal-Driven Staged Rehabilitation	Breaks down dysphagia rehabilitation into step-by-step motor objectives (e.g., transitioning from passive sensory stimulation to active functional swallowing).	Nurse-led
da Silva (26) (2018) Brazil	SLP-Integrated Care Model	Elevates swallowing management from routine generalized care to specialized interventions, ensuring standardized diet modification and targeted muscle therapy.	SLP-led

#### 3.6.2 Implementing Personnel for Interventions

Six of eight interventional studies explicitly reported intervention personnel. Da Silva et al. (26) was fully SLP-led. Mo et al. (14) employed dual delivery by rehabilitation therapists and nurses. Zhou et al. (17) built a multidisciplinary team. Remaining studies used nurses as primary implementers (16, 19, 20). Two studies did not specify the professional background of implementers (Table 5).

**Table 5:** Summary of interventional studies for pediatric post-extubation dysphagia.

Author	n	Intervention focus & dosage (freq/duration)	Implementer	Primary outcomes
Chen (16) (2025) China	112	Focus: OMI (acupoint/oral massage, palate and gum stimulation). Dosage: 1 h before feeding daily, for 4 wk.	Nurses	Dysphagia symptom improvement, growth indices, FLACC score
Mo (14) (2024) China	120	Focus: OMI (oral massage, pharyngeal sensory stimulation, neck mobility) + NNS (pacifier). Dosage: OMI 15 min (2x/d); NNS 5 min (6x/d).	Rehab therapists, Nurses	SSA score, dysphagia improvement rate, complication rate
Li (18) (2023) China	100	Focus: Targeted oral muscle training, progressive feeding interventions. Dosage: 20 min/session (2x/d).	Not specified	SSA, DOSS, dysphagia recovery time
Zhou (17) (2023) China	95	Focus: Jaw/lip stimulation, caregiver education. Dosage: Before meals, for 1 mo.	Multidisciplinary nursing team	NOMAS, NGT duration, FLACC, sleep quality
Liu Q (20) (2022) China	60	Focus: OMI (cheek/tongue massage, jaw support) + NNS. Dosage: 12 min/session (3x/d; 30 min before feeding).	Specialized intervention team	NOMAS, NGT duration, time to full oral feeding
Liu L (19) (2022) China	100	Focus: Ice stick sensory stimulation, NMES, acupoint massage, semi-recumbent feeding. Dosage: Ice/NMES 20 min/session (2x/d) for 2 wk.	Nurses	Dysphagia improvement rate, choking frequency, NGT duration
Zhang (15) (2019) China	80	Focus: OMI (C-shaped oral massage, soft palate/tongue stimulation) + NNS. Dosage: 15−20 min/session (3x/d; 0.5 h before feeding).	Not specified (only medical staff)	SSB coordination, time to full oral feeding
daSilva (26) (2018) Brazil	115	Focus: Diet texture/viscosity modification, compensatory maneuvers, swallowing muscle strengthening. Dosage: 30−45 min (1x/d) until dysphagia resolved.	SLP	Time to initiate/reach full oral feeding, complication rate (e.g., pneumonia)

OMI, oral motor intervention; NNS, non-nutritive sucking; NMES, neuromuscular electrical stimulation; SLP, speech-language pathologist; NGT, nasogastric tube; SSB, suck–swallow–breathe coordination; FLACC, Face Legs Activity Cry Consolability scale; SSA, Standardized Swallowing Assessment; NOMAS, Neonatal Oral-Motor Assessment Scale; DOSS, Dysphagia Outcome and Severity Scale.

#### 3.6.3 Intervention Technique Combinations

Seven of eight interventional studies involved oral motor training. Technique frequency: oral massage and active movement training of the cheeks, perioral region, tongue, and mandible (*n* = 6) (14–17, 19, 20); non-nutritive sucking (*n* = 4) (14–16, 20); feeding training including positional adjustment, progressive texture transition, and compensatory swallowing maneuvers (*n* = 3) (14, 17, 19); oral sensory stimulation combining thermal and mucosal stimulation (*n* = 2) (15, 19); traditional Chinese medicine acupoint massage (*n* = 2) (16, 19); pharyngeal sensory stimulation plus cervical range-of-motion training (*n* = 1) (14); and neuromuscular electrical stimulation (NMES) (*n* = 1) (19). Chen & Lou (16) targeted PICU infants aged 36–41 gestational weeks over a 4-week intervention, using not only NOMAS scores but also weight, length, chest circumference, and SGNA nutritional scores as outcomes. Details are presented in Table 5.

#### 3.6.4 Outcome measures

Primary outcome measures across eight interventional studies: swallowing function scale scores (SSA, NOMAS, DOSS, FOIS) in all eight studies (14–20, 26); time to full oral feeding or NGT dwell duration in five (15, 16, 19, 20, 26); length of hospital stay in seven (14–17, 19, 20, 26); NGT placement duration in five (16–19, 26); complication rates (aspiration, aspiration pneumonia, re-intubation) in four (14, 18, 19, 26). All studies reported improvements in the intervention group on their respective primary measures (Table 5).

## 4 Discussion

### 4.1 Disease burden and developmental specificity

None of the included studies validated their bedside assessment instruments against VFSS or FEES. Reported incidence figures therefore reflect clinically apparent dysphagia only and cannot account for silent aspiration, a limitation relevant to all findings discussed below. The reported incidence of PED in critically ill children ranges from 28.36% to 68.94% (5, 8–13). This wide variation reflects methodological heterogeneity across studies: inconsistent diagnostic criteria (differing scale cut-offs and clinical sign definitions), variable assessment timing (4–72 h post-extubation), differences in assessor professional background (SLP versus nurse), and variation in disease composition of study populations. These factors limit cross-study comparisons.

All included studies used bedside clinical scales; none introduced FEES or VFSS. The reported figures therefore reflect clinical apparent status only and cannot detect silent aspiration (29, 64). This limitation carries particular clinical weight in children. In adult critical care, Bricker et al. (7) found that silent aspiration affected 78% of clinically stable post-extubation patients on instrumental assessment. No equivalent instrumental cohort data exist for children. Because infants and young children have a higher cough reflex threshold and incompletely developed protective airway reflexes (30), silent aspiration is even harder to identify through routine clinical signs, and the true incidence of pediatric PED likely exceeds available estimates.

With respect to structural injury, Dutta et al. (31) performed endoscopic screening in 122 children and found approximately 39.3% had positive findings such as subglottic stenosis or intubation granulomas, suggesting laryngopharyngeal structural injury after intubation may constitute an important pathological basis for pediatric PED.

In sum, pediatric PED faces multiple interrelated challenges: limited assessment methods, objective laryngopharyngeal structural pathology, and developmental specificity that prevents direct translation of adult diagnostic standards and management strategies. These factors collectively make diagnosing and managing PED in children more complex than in adults, underscoring the need for pediatric-specific research.

### 4.2 Risk factor identification and prediction model development

#### 4.2.1 Evidence integration for core predictors and pathophysiological mechanisms of emerging predictors

Among the included studies, intubation duration was reported in five studies, age in four studies, and IWS in three studies (Tier 1). Neurological comorbidities and number of intubations (≥2 times) were each reported in two studies (Tier 2). IWS was first incorporated into multivariable regression by da Silva et al. (5) and was the most heavily weighted predictor in the CRISPED score; Zhu et al. (9) also reported IWS as a predictor in their multivariable model. Delirium was reported in one single-center study (11) (Tier 3).

Regarding pathophysiology, a plausible mechanism for repeated intubations as a predictor is that cumulative mechanical trauma to the sensory branches of the superior laryngeal nerve may progressively lower the swallowing reflex threshold; Dutta et al. (31) found positive endoscopic lesions in approximately 39.3% of children. The mechanism linking delirium to dysphagia has been explored in adult critical care, though not in the included pediatric studies: In adult critical care, ICU delirium has been associated with impaired cortical control over pharyngeal swallowing initiation and airway protection (3, 32); whether a comparable mechanism operates in the developing brain, where cortical control over swallowing remains immature (33, 34), warrants prospective investigation.

#### 4.2.2 Dual effects of the endotracheal tube cuff

Da Silva et al. (5) found cuffed tubes associated with reduced PED risk (OR=0.35, 95% CI 0.13–0.95), possibly because better subglottic sealing reduces micro-aspiration and unplanned tube exchange; in contrast, Dutta et al. (31) found a markedly higher rate of positive endoscopic lesions in the cuffed tube group (84.2%, 32/38) compared with the uncuffed group (19.0%, 16/84; *P* < 0.0001), implying that sustained radial cuff pressure can cause local ischemia and granuloma formation. Two key confounders explain this apparent paradox: different assessment windows (24–48 h vs. 3–6 months post-extubation) and different patient populations (uncuffed tubes predominantly used in younger children, who carry higher baseline PED risk). Intubation duration, a recognized PED risk factor, was not a central analytical variable in either study, but its potential interaction with cuff effects merits attention.

These two studies differ substantially in design, time horizon, and patient population and should not be compared directly. A working hypothesis can be proposed: the effect of cuffed tubes on post-extubation outcomes may be time-dependent—in the early post-extubation period, the primary cuff effect may be reduction of micro-aspiration, lowering short-term PED risk; in the late post-extubation period, the primary risk may shift to pressure-induced mucosal injury. This hypothesis warrants testing in prospective studies specifically designed to control for age confounding, with tube physical properties included as independent variables in future prediction model development.

#### 4.2.3 Methodological strengths and limitations of the CRISPED score

Over nearly a decade of systematic inquiry, the da Silva team has built a coherent evidence chain: SLP-led intervention exploration (26), prospective risk factor identification (5), and CRISPED score development and validation (10). The CRISPED score is the only pediatric PED prediction tool with temporal internal validation. Its methodological strengths include use of LASSO regression to control overfitting and restriction to three variables obtainable at the bedside. The three-tier risk stratification provides an actionable basis for resource allocation: high-risk patients receive prioritized SLP evaluation, while low-risk patients avoid unnecessary intervention—particularly meaningful given universal SLP scarcity in the PICU.

Key limitations reported by the original authors include: single-center design with limited representativeness; diagnosis based on SLP bedside assessment without an instrumental gold standard, introducing possible misclassification bias; study population composed mainly of medical patients, with high-risk surgical patients excluded; and the absence of any multicenter external validation. Despite these limitations, CRISPED's concise design, good discrimination, and clear risk stratification offer important clinical reference value, pending confirmation from independent multicenter validation.

### 4.3 Assessment tools, interventions, and methodological limitations

#### 4.3.1 Current state of assessment tools

None of the included instruments was validated against VFSS or FEES as a reference standard. Reported sensitivity and specificity estimates therefore reflect performance against clinical composite assessments rather than instrumental gold standards, leaving the true diagnostic gap unquantified. In the adult field, instruments such as the Yale Swallow Protocol and GUSS-ICU have undergone full validation including concurrent validity against FEES, multicenter reliability and validity testing, and predictive validity for clinical outcomes (35–37). Pediatric tools lag substantially behind these standards. Adult PED is primarily associated with neuromuscular dysfunction or iatrogenic factors; in children, developmental immaturity, small anatomical structures, and low cooperativeness add complexity (38). Among tools used in included studies, FOIS-based bedside functional assessment cannot detect silent aspiration; water-swallow tests (SSA, MWST) require volumes exceeding safe capacities for infants and young children, lack standardized pediatric protocols, and depend on active cooperation, making reliability uncertain in this population.

Chinese investigators have begun addressing these limitations. Hu (11) developed a pediatric adaptation of the adult GUSS-ICU (P-GUSS-ICU) applicable to children aged 3–14 years. Wang (13) developed a 10-item dual-dimension scale (PED-C) for children aged 2–14 years. Both tools are designed for the PICU environment, rely on external observation suitable for patients with limited verbal expression, and have reported acceptable reliability and validity. However, both have been validated only at a single center, leaving applicability across different resource levels untested; both cover children aged 2 or 3 years and above, leaving infants under 24 months inadequately covered; and both used clinical composite assessments rather than VFSS or FEES as reference standards, leaving the true diagnostic gap against the gold standard unquantified.

#### 4.3.2 Instrumental assessment constraints and the circular validation problem

Although VFSS and FEES are recognized reference standards for diagnosing silent aspiration and structural injury (29), PICU implementation is constrained. VFSS involves intra-hospital transport risk, radiation exposure, and the risk of barium aspiration, making it unsuitable for hemodynamically unstable patients in the early post-extubation period (39). FEES can be performed at the bedside but its invasiveness may injure the fragile, narrow nasopharyngeal mucosa of infants and young children (40); its concordance with VFSS is also lower in children than in adults (40). Both procedures require specialized equipment and trained personnel in pediatric developmental swallowing, a specialty with limited staffing (41).

These practical constraints have driven researchers to use clinical screening tools as substitute reference standards rather than VFSS or FEES. However, substitute reference standards differ fundamentally from gold standards in their diagnostic basis. When a screening tool serves as the criterion, the true diagnostic gap between a new tool and the gold standard cannot be accurately quantified, and estimates of sensitivity and specificity may be biased. More critically, if the criterion tool itself cannot detect silent aspiration, a new scale validated against it will share this same blind spot. This circular validation cycle—validating one screening tool against another without access to gold-standard diagnostic capability—is the key methodological obstacle limiting progress in the field. No effective solution to this cycle currently exists in the pediatric PED assessment tool literature.

#### 4.3.3 Objective assessment technologies and alternative validation strategies

Two complementary directions have emerged. At the technological level, Chen et al. (42) developed and validated a nurse-led bedside diagnostic model based on acoustic analysis of cervical tracheal breath sounds, using an electronic stethoscope to record pre- and post-swallow respiratory sounds and extract objective acoustic parameters (derivation AUC 0.785; validation AUC 0.746). This non-invasive approach, requiring no imaging exposure or nasal insertion, provides an alternative route to objective silent aspiration detection. At the evaluation level, da Silva et al. (10) incorporated decision curve analysis in CRISPED development, showing that clinical benefit from CRISPED-guided intervention decisions exceeded that of treating all or treating none across the clinically relevant 20%–60% probability threshold range. This approach delineates the useful application range of the tool and offers a methodological reference for assessing clinical utility under resource constraints.

#### 4.3.4 Heterogeneity of assessment timing and evidence gaps

Across included studies, the first assessment time point ranged from 4 to 72 h post-extubation (5, 8–15, 26); no study compared different timing approaches on diagnostic accuracy or clinical outcomes. In the adult field, Leder et al. (43) found that 82.2% of patients passed a swallowing screen at 1 h post-extubation, and delayed assessment did not reduce aspiration risk; Marvin et al. (44) showed by FEES that 69% of patients could safely swallow at least one food texture at 2–4 h post-extubation. In the pediatric PICU context, early assessment faces distinct challenges: residual sedation may impair assessment accuracy—within 4 h of extubation, children may still have circulating sedative metabolites (45) and may not reach the alertness required for assessment. Young children cannot perform the command-based tasks on which adult screening protocols depend (46), and age-appropriate preconditions for assessment remain unstandardised across paediatric age groups (47). Determining optimal timing will require prospective studies designed specifically for this population.

#### 4.3.5 Resource allocation for intervention personnel and strategic integration

McRae et al. (48) identify SLPs as the core specialist for ICU dysphagia management. Da Silva et al. (26) reported that integrating SLPs into routine PICU management was associated with a reduction in re-intubation rates from 24.3% to 2.7%. However, SLP resources are severely unequal globally: Wylie et al. (49) report SLP-to-population ratios of 1:2,500–1:4,700 in high-income countries and as low as 1:2,000,000–1:4,000,000 in four sub-Saharan African countries. Given this reality, evidence from adult stroke and ICU populations suggests that nurse-led screening may be feasible: Hinchey et al. (50) showed in a multicenter study that nurse-led formal swallowing screening protocols reduced aspiration pneumonia incidence from 5.4% to 2.4%; Palli et al. (51) demonstrated similar associations; Macht et al. (52) argued that nurse-executed front-line screening is a prerequisite for rational SLP resource allocation. Whether these findings translate to the pediatric PICU requires prospective evaluation. This review suggests a nurse-led multidisciplinary collaborative system as a direction worth prospective testing: nurses conduct standardized screening and risk stratification; SLPs focus on confirmatory diagnosis and individualized rehabilitation in high-risk patients; physicians oversee overall treatment decisions. The nurse role is strictly limited to early screening and risk triage—it does not substitute for SLP diagnostic functions.

Regarding intervention strategies, current studies rely primarily on oral motor training. Studies differ markedly in training frequency, session duration, total intensity, initiation timing, and stopping criteria, making evidence synthesis difficult. Diet texture modification, environmental adaptation, and preventive oral care have not been systematically incorporated. Compared with international frameworks such as those from the Royal College of Speech and Language Therapists (48), current studies show a clear gap in operational standardization and multidimensional integration, further underscoring the need to establish a core outcome set for PED in critically ill children (53).

#### 4.3.6 Special considerations for infants and young children

Included studies consistently identify young age as an independent risk factor. Beyond risk magnitude, young age also raises questions about the applicability of existing assessment and intervention approaches. Regarding differential diagnosis, Jadcherla (54) notes that infant feeding difficulties are influenced by developmental immaturity and incompletely established suck–swallow–breathe coordination reflexes (55); the clinical presentation of intubation-related swallowing injury overlaps substantially with developmental feeding intolerance and gastroesophageal reflux, and available tools have not systematically incorporated differential diagnosis modules for these confounders.

Regarding intervention goals, older children require restoration of a previously established function, while infants’ suck–swallow–breathe rhythm is itself still developing; intubation-related sensorimotor interruption may impair normal acquisition of this rhythm, so the intervention target involves restarting a developmental process rather than simple functional recovery. At the clinical implementation level, most current approaches use rehabilitation strategies designed for active cooperation, which are difficult to implement effectively in infants; passive techniques should be prioritized. Some guidelines reference air-jet sensory training (56, 57); non-nutritive sucking has a documented application basis in preterm infant feeding management (58). However, prospective evidence for the efficacy and safety of both techniques in critically ill infants with PED is lacking. Chen & Lou (16) is the only study that included nutritional and growth developmental indices as co-primary outcomes. Infants and very young children should be treated as an independent research population rather than as a subgroup within broader pediatric studies.

### 4.4 Geographic concentration of evidence and divergent research pathways

Of the 15 included studies, 10 originated from mainland China, 3 from Brazil, and only 2 from high-income countries (8, 12). High-income country studies (USA and Japan) are predominantly retrospective cohorts, report incidence of 29.03% to 41.51% (8, 12), and include few interventional studies. China (population approximately 1.41 billion (59); GDP approximately USD 18.75 trillion (60) and Brazil (population approximately 216 million (61); GDP approximately USD 2.19 trillion (60) have produced active, application-driven research output (5, 9–11, 13–20, 26). Large PICU patient volumes in both countries make pediatric PED a clinically prominent problem. Chinese studies are characterized by rapid production spanning scale development, intervention technique combinations, and management pathway construction (11, 13–20), with fast clinical translation but insufficient standardization. The Brazilian da Silva team completed a coherent evidence chain from risk factor identification to intervention exploration to prediction model development and validation within one institution (5, 10, 26), demonstrating strong methodological continuity but with generalizability limited by single-center design.

Study designs also differ markedly by country: Chinese studies are predominantly quasi-experimental or retrospective, while the Brazilian studies include a prospective cohort and an intervention study with a historical control group; the two high-income country studies are both retrospective cohorts. The divergent research emphases across these settings reflect structural differences in healthcare systems. In the included Chinese studies, rehabilitation therapists and nurses served as primary assessors and intervention implementers in all cases, suggesting limited SLP involvement in routine PICU swallowing assessment in these settings, which explains the strong emphasis on nurse-administered scale development and nurse-led intervention protocols. The Brazilian da Silva team's coherent evidence chain was conducted in a tertiary academic PICU with established SLP integration (5, 10, 26), a resource context that differs substantially from the average Chinese PICU setting. These structural differences may also contribute to incidence variation across studies: SLP-administered assessments such as P-FOIS may detect mild dysphagia more sensitively than nurse-administered tools. Key outcome measures also vary by setting: Chinese studies predominantly report swallowing scale scores and feeding tube duration, while Brazilian studies report functional oral intake and re-intubation rates. Future cross-cultural studies should explicitly describe local SLP availability, assessor training, and standard PED management processes to enable meaningful comparison. Future Chinese research should maintain its application-driven advantage while learning from the methodological rigor of Brazilian and high-income country approaches, with multicenter collaboration across underrepresented regions as a priority.

### 4.5 A proposed conceptual framework for future research: NRMPF-PED

Based on the evidence gaps identified in this review, we propose the Nurse-led Risk-stratified Management Framework for Post-extubation Dysphagia (NRMPF-PED) as a conceptual model intended to generate hypotheses for future implementation science research, not as a clinical pathway ready for practice. The framework conceptualizes a shift from passive post-extubation assessment to proactive pre-extubation risk surveillance, with nurse-led screening combined with targeted multidisciplinary responses. All components described below are extrapolated from the current evidence base and require prospective validation before any clinical application. The NRMPF-PED comprises five sequential components.

Component 1 – Pre-intubation risk assessment: in this proposed component, initial patient risk information (age, growth parameters, baseline neurological status) would be collected before intubation to inform tube selection decisions and initiate cuff-pressure monitoring where applicable.

Component 2 – Bundle-based prevention during intubation: this component proposes integrating analgesia-sedation management, daily awakening assessments, delirium screening (62,63,64), and preventive oral care into routine intubation care, with systematic documentation of intubation episodes and ventilation duration as core CRISPED score variables. The feasibility of this component in pediatric settings requires prospective evaluation, as the underlying evidence is extrapolated from adult critical care literature.

Component 3 – Initial risk flagging at extubation: this component proposes using a partial CRISPED score (number of intubations and intubation days) at the point of extubation to enable early resource allocation. This partial score has not been validated (10) and would serve only as a preliminary reference; it could not substitute for the complete validated score. Whether this approach improves outcomes requires prospective testing.

Component 4 – Dual post-extubation assessment: this component proposes combining IWS-based completion of the full CRISPED score (10) with concurrent Wang scale (13) bedside screening to generate a risk stratification result. The Wang scale's limited applicability in infants under 24 months is acknowledged; alternative approaches such as suck–swallow–breathe coordination observation would need to be evaluated for this age group. How these two tools should be combined in practice requires prospective implementation research.

Component 5 – Risk-stratified multidisciplinary response: this component proposes that risk stratification results from Component 4 would guide differentiated resource allocation, with lower-risk patients receiving nurse-led preventive care and higher-risk patients receiving prioritised SLP involvement. The specific thresholds, response protocols, and monitoring intervals described in this conceptual model are hypothetical and would need to be defined and tested in prospective implementation studies before any clinical application.

**Figure 2:**
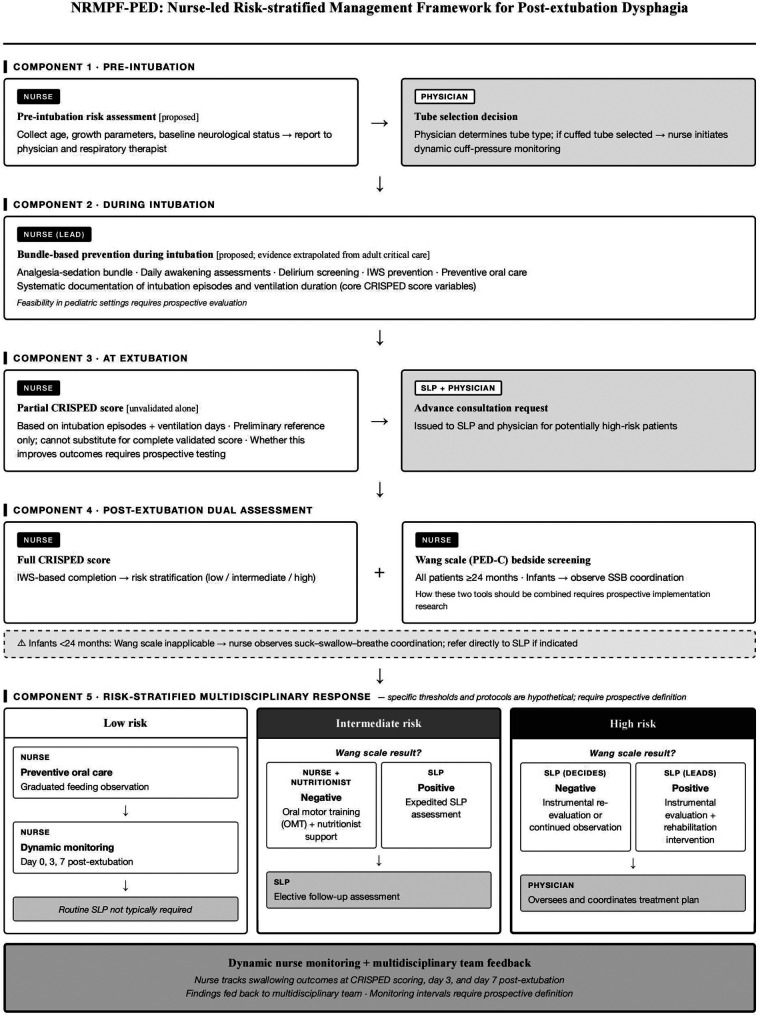
Conceptual diagram of the nurse-led risk-stratified management framework for post-extubation dysphagia (NRMPF-PED). The framework comprises five sequential components: (1) pre-intubation risk assessment, (2) dynamic bundle management during intubation, (3) initial risk flagging at extubation, (4) dual post-extubation assessment (CRISPED score + Wang scale), and (5) stratified multidisciplinary response. NRMPF-PED is a hypothetical conceptual model for future research; all five components require prospective implementation science research before any clinical application, and this framework does not constitute a clinical practice guideline.

The NRMPF-PED is a research hypothesis, not a practice guideline. Key limitations include: the partial CRISPED score in Component 3 is unvalidated; the Wang scale has limited applicability in infants under 24 months; Component 2 strategies are extrapolated from adult critical care literature; and the multidisciplinary mechanisms in Components 4 and 5 reflect expert inference rather than pediatric evidence. Prospective implementation science research and independent ethics review are required before any operationalization Figure 2 – NRMPF-PED Framework Diagram.

## 5 Limitations

This review has six main limitations. First, only 15 studies were included, with pronounced geographic concentration in mainland China (*n* = 10) and Brazil (*n* = 3); very sparse evidence from high-income Western countries restricts global generalizability. The reasons for this concentration are discussed in Section 4.4, but the geographic imbalance remains, limiting the global applicability of the conclusions. Second, substantial heterogeneity across studies in PED diagnostic criteria, assessment tools, participant characteristics, and definitions of mechanical ventilation duration precluded quantitative synthesis; meta-analysis was not feasible. Third, in keeping with Arksey and O'Malley's scoping review framework as updated by JBI guidelines, no formal risk-of-bias assessment was performed and no certainty-of-evidence ratings were assigned. Fourth, the search cutoff was mid-March 2026, and studies published after that date are not included. Fifth, the NRMPF-PED conceptual management framework is a theoretical construct based on current evidence and has not been tested in prospective implementation research. Sixth, two included studies are master's theses; findings from these should be interpreted with greater caution than journal-published data.

## 6 Conclusions

This scoping review maps current evidence on PED in critically ill children. None of the included bedside assessment instruments was validated against VFSS or FEES; reported incidence and accuracy data consequently reflect clinical apparent status only. Establishing instrumental validity for bedside tools represents the most pressing methodological gap in the field. Incidence estimates range from 28.36% to 68.94%, with cross-study variation largely attributable to heterogeneity in diagnostic criteria, assessment timing, and assessor background. Risk factors reported across multiple independent multivariable models are summarised using the three-tier framework described in the Results. The CRISPED score is the only prediction tool with temporal internal validation; external validation is lacking. Intervention protocols vary substantially in design, dosage, and outcome measurement, precluding synthesis. Evidence specific to infants under 12 months remains sparse. The included studies originate predominantly from China and Brazil, which limits generalisability.

Key research priorities include: (1) multicenter external validation of the CRISPED score; (2) development of age-appropriate bedside screening tools with concurrent validity against VFSS or FEES, with particular attention to infants under 6 months; (3) establishment of a core outcome set to standardise diagnostic criteria and assessment timing; (4) age-stratified trials to define optimal parameters for oral motor training; (5) prospective evaluation of nurse-led screening and risk-stratification pathways; (6) multiregional studies to test the cross-cultural applicability of existing tools and protocols.

## Data Availability

All data supporting the conclusions of this study are presented in the main text and appendices. Detailed data extraction forms are available from the corresponding author on request.

## Glossary

AIS: Abbreviated Injury Scale
AUC: Area Under the Curve
CBM: Chinese Biomedical Literature Database
CI: Confidence Interval
CNKI: China National Knowledge Infrastructure
CPG: Central Pattern Generator
CRISPED: Clinical Risk Score for Post-extubation Dysphagia
DOSS: Dysphagia Outcome and Severity Scale
EPV: Events Per Variable
ETT: Endotracheal Tube
FEES: Fiberoptic Endoscopic Evaluation of Swallowing
FIDS: Feeding Interaction and Development Scale
FLACC: Face, Legs, Activity, Cry, Consolability Scale
FOIS: Functional Oral Intake Scale
GERD: Gastroesophageal Reflux Disease
GRADE: Grading of Recommendations, Assessment, Development and Evaluations
GUSS-ICU: Gugging Swallowing Screen – Intensive Care Unit
IWS: Iatrogenic Withdrawal Syndrome
JBI: Joanna Briggs Institute
MWST: Modified Water Swallowing Test
NGT: Nasogastric Tube
NMBA: Neuromuscular Blocking Agent
NMES: Neuromuscular Electrical Stimulation
NNS: Non-Nutritive Sucking
NOMAS: Neonatal Oral-Motor Assessment Scale
NRMPF-PED: Nurse-led Risk-stratified Management Framework for Post-extubation Dysphagia
OMI: Oral Motor Intervention
OR: Odds Ratio
OSF: Open Science Framework
P-FOIS: Pediatric Functional Oral Intake Scale
P-GUSS-ICU: Pediatric Gugging Swallowing Screen – Intensive Care Unit
PCC: Population, Concept, Context
PED: Post-Extubation Dysphagia
PED-C: Post-Extubation Dysphagia in Children Screening Tool
PICU: Pediatric Intensive Care Unit
PRISMA-ScR: Preferred Reporting Items for Systematic Reviews and Meta-Analyses – Scoping Reviews Extension
RASS: Richmond Agitation–Sedation Scale
RCT: Randomized Controlled Trial
RE-AIM: Reach, Effectiveness, Adoption, Implementation, Maintenance Framework
SGNA: Subjective Global Nutritional Assessment
SLP: Speech-Language Pathologist
SOS: Sophia Observation Withdrawal Symptoms Scale
SSA: Standardized Swallowing Assessment
SSB: Suck–Swallow–Breathe Coordination Scale
TF: Trial Feeding
VIP: VIP Chinese Journal Service Platform.
