# Supplementary material for: Characteristics, predictors, and management of post-extubation dysphagia in critically Ill children: a scoping review
Source: Front Pediatr. 2026 Jun 15;14:1854127. doi: 10.3389/fped.2026.1854127 (PMC13311104; doi:10.3389/fped.2026.1854127)
Supplement: Supplementary file 1 [file Table1.docx]

S1: Search Process

PubMed

Data searched: 12 March 2026

| **#** | **Search terms** | **Result retrieved** |
| --- | --- | --- |
| 1 | ("Child"[Mesh] OR "Child, Preschool"[Mesh] OR "Adolescent"[Mesh] OR "Pediatrics"[Mesh]) | 3650287 |
| 2 | (child[Title/Abstract] OR children[Title/Abstract] OR preschool child*[Title/Abstract] OR adolescent*[Title/Abstract] OR adolescence[Title/Abstract] OR teenager*[Title/Abstract] OR youth*[Title/Abstract] OR teen*[Title/Abstract] OR pediatric*[Title/Abstract] OR paediatric*[Title/Abstract]) | 2272867 |
| 3 | 1 OR 2 | 4337316 |
| 4 | ("Intubation"[MeSH Terms] OR "Airway Extubation"[MeSH Terms] OR "respiration, artificial"[MeSH Terms]) | 149138 |
| 5 | ("extubation"[Title/Abstract] OR "postextubation"[Title/Abstract] OR "post-extubation"[Title/Abstract] OR "Intubation"[Title/Abstract] OR "Airway Extubation"[Title/Abstract] OR "Respiration, Artificial"[Title/Abstract] OR "after extubation"[Title/Abstract]) | 79054 |
| 6 | 4 OR 5 | 192245 |
| 7 | ("Deglutition Disorders"[MeSH Terms]) | 64596 |
| 8 | ("oropharyngeal dysphagia"[Title/Abstract] OR "esophageal dysphagia"[Title/Abstract] OR "swallow* dysfunction"[Title/Abstract] OR "swallow* impair*"[Title/Abstract] OR "swallow* disord*"[Title/Abstract] OR "deglutition dysfunction"[Title/Abstract] OR "deglutition impair*"[Title/Abstract] OR "deglutition disorder*"[Title/Abstract] OR "acquired dysphagia"[Title/Abstract] OR "dysphagia*"[Title/Abstract] OR "impaired swallowing"[Title/Abstract] OR "swallow problem*"[Title/Abstract] OR "Esophageal Motility Disorders"[Title/Abstract] OR "swallowing disturbance"[Title/Abstract] OR "swallowing difficult"[Title/Abstract]) | 45647 |
| 9 | 7 OR 8 | 88787 |
| 10 | 3 AND 6 AND 9 | 512 |

EMBASE

Data searched: 12 March 2026

| **#** | **Search terms** | **Result retrieved** |
| --- | --- | --- |
| 1 | 'child'/exp OR 'preschool child'/exp OR 'adolescent'/exp OR 'pediatrics'/exp | 4977598 |
| 2 | 'child':ad,ti OR 'children':ad,ti OR 'preschool child*':ad,ti OR 'adolescent*':ad,ti OR 'adolescence':ad,ti OR 'teenager*':ad,ti OR 'youth*':ad,ti OR 'teen*':ad,ti OR 'pediatric*':ad,ti OR 'paediatric*':ad,ti | 3608728 |
| 3 | #1 OR #2 | 6322294 |
| 4 | 'intubation'/exp OR 'extubation'/exp OR 'artificial ventilation'/exp | 478714 |
| 5 | 'extubation':ad,ti OR 'postextubation':ad,ti OR 'post-extubation':ad,ti OR 'intubation':ad,ti OR 'airway extubation':ad,ti OR 'respiration, artificial':ad,ti OR 'after extubation':ad,ti | 30310 |
| 6 | #4 OR #5 | 480527 |
| 7 | 'dysphagia'/exp | 130458 |
| 8 | 'oropharyngeal dysphagia':ad,ti OR 'esophageal dysphagia':ad,ti OR 'swallow* dysfunction':ad,ti OR 'swallow* impair*':ad,ti OR 'swallow* disord*':ad,ti OR 'deglutition dysfunction':ad,ti OR 'deglutition impair*':ad,ti OR 'deglutition disorder*':ad,ti OR 'acquired dysphagia':ad,ti OR 'dysphagia*':ad,ti OR 'impaired swallowing':ad,ti OR 'swallow problem*':ad,ti OR 'esophageal motility disorders*':ad,ti OR 'swallowing disturbance':ad,ti OR 'swallowing difficult':ad,ti | 19785 |
| 9 | #7 OR #8 | 132053 |
| 10 | #3 AND #6 AND #9 | 1604 |

Web of Science

Data searched: 12 March 2026

| **#** | **Search terms** | **Result retrieved** |
| --- | --- | --- |
| 1 | TS=("child" OR "child,preschool" OR "adolescent" OR "pediatrics" OR "children" OR "preschool child*" OR "adolescent*" OR "adolescence" OR "teenager*" OR "youth*" OR "teen*" OR "pediatric*" OR "paediatric*" ) | 2708643 |
| 2 | TS=("Intubation" OR "Airway Extubation" OR "Respiration, Artificial" OR "extubation" OR "postextubation" OR "post-extubation" OR "following extubation" OR "after extubation") | 61628 |
| 3 | TS=("Deglutition Disorders"OR "oropharyngeal dysphagia" OR "esophageal dysphagia" OR "swallow* dysfunction" OR "swallow* impair*" OR "swallow* disord*" OR "deglutition dysfunction" OR "deglutition impair*" OR "Deglutition Disorder*" OR "acquired dysphagia" OR "Dysphagia*" OR "impaired swallowing" OR "swallow problem*" OR "Esophageal Motility Disorders" OR "swallowing disturbance" OR "swallowing difficult") | 39753 |
| 4 | 1 AND 2 AND 3 | 117 |

Cochrane Library

Data searched: 12 March 2026

| **#** | **Search terms** | **Result retrieved** |
| --- | --- | --- |
| 1 | MeSH descriptor: [Child] explode all trees | 84340 |
| 2 | MeSH descriptor: [Pediatrics] explode all trees | 1076 |
| 3 | MeSH descriptor: [Adolescent] explode all trees | 139887 |
| 4 | MeSH descriptor: [Child, Preschool] explode all trees | 40936 |
| 5 | (child):ti,ab,kw OR (children):ti,ab,kw OR (preschool child*):ti,ab,kw OR (adolescent*):ti,ab,kw OR (adolescence):ti,ab,kw OR (teenager*):ti,ab,kw OR (youth*):ti,ab,kw OR (teen*):ti,ab,kw OR (pediatric*):ti,ab,kw OR (paediatric*):ti,ab,kw | 337396 |
| 6 | #1 OR #2 OR #3 OR #4 OR #5 | 337462 |
| 7 | MeSH descriptor: [Deglutition Disorders] explode all trees | 4282 |
| 8 | (oropharyngeal dysphagia):ti,ab,kw OR (esophageal dysphagia):ti,ab,kw OR (swallow* dysfunction):ti,ab,kw OR (swallow* impair*):ti,ab,kw OR (swallow* disord*):ti,ab,kw OR (deglutition dysfunction):ti,ab,kw OR (deglutition impair*):ti,ab,kw OR (Deglutition Disorder*):ti,ab,kw OR (acquired dysphagia):ti,ab,kw OR (Dysphagia*):ti,ab,kw OR (impaired swallowing):ti,ab,kw OR (swallow problem*):ti,ab,kw OR (Esophageal Motility Disorders*):ti,ab,kw OR (swallowing disturbance):ti,ab,kw OR (swallowing difficult):ti,ab,kw | 8291 |
| 9 | #7 OR #8 | 10735 |
| 10 | MeSH descriptor: [Airway Extubation] explode all trees | 483 |
| 11 | MeSH descriptor: [Intubation] explode all trees | 6841 |
| 12 | MeSH descriptor: [Respiration, Artificial] explode all trees | 9582 |
| 13 | (extubation):ti,ab,kw OR (postextubation):ti,ab,kw OR (post-extubation):ti,ab,kw OR (intubation):ti,ab,kw OR (airway extubation):ti,ab,kw OR (after extubation):ti,ab,kw OR (following extubation):ti,ab,kw OR (mechanical ventilation):ti,ab,kw | 8291 |
| 14 | #10 OR #11 OR #12 OR #14 | 23460 |
| 15 | #6 AND #9 AND #14 | 1001 |

CINAHL

Data searched: 12 March 2026

| **#** | **Search terms** | **Result retrieved** |
| --- | --- | --- |
| 1 | XB ("child" OR "child,preschool" OR "adolescent" OR "pediatrics" OR "children" OR "preschool child*" OR "adolescent*" OR "adolescence" OR "teenager*" OR "youth*" OR "teen*" OR "pediatric*" OR "paediatric"*) | 824411 |
| 2 | XB ("Intubation" OR "Airway Extubation" OR "Respiration, Artificial" OR "extubation" OR "postextubation" OR "post-extubation" OR "following extubation" OR "after extubation") | 22140 |
| 3 | XB ("Deglutition Disorders"OR "Esophageal Motility Disorders" OR "oropharyngeal dysphagia" OR "esophageal dysphagia" OR "swallow* dysfunction" OR "swallow* impair*" OR "swallow* disord*" OR "deglutition dysfunction" OR "deglutition impair*" OR "Deglutition Disorder*" OR "acquired dysphagia" OR "Dysphagia*" OR "impaired swallowing" OR "swallow problem*" OR "Esophageal Motility Disorders" OR "swallowing disturbance" OR "swallowing difficult") | 12564 |
| 4 | 1 AND 2 AND 3 | 24 |

CNKI

Data searched: 12 March 2026

| **#** | **Search terms** | **Result retrieved** |
| --- | --- | --- |
| 1 | (篇关摘：儿童 + 儿科 + 患儿 + 幼儿 + 小儿 + 青少年 (精确)) | 1941663 |
| 2 | (篇关摘：气管插管 + 人工气道 + 人工通气 + 机械通气 + 气管拔管 + 拔管(精确)) | 153012 |
| 3 | (篇关摘：吞咽困难 + 吞咽障碍 + 口咽吞咽困难 + 进食困难 + 咽下困难 + 拔管后吞咽困难 + 获得性吞咽困难 + 获得性吞咽障碍(精确)) | 31793 |
| 6 | 1 AND 2 AND 3 | 41 |

VIP

Data searched: 12 March 2026

| **#** | **Search terms** | **Result retrieved** |
| --- | --- | --- |
| 1 | 摘要=儿童 OR 儿科学 OR 儿科 OR 患儿 OR 小儿 OR 青少年 | 1258954 |
| 2 | 摘要=气管插管 OR 人工气道 OR 人工通气 OR 机械通气 OR 气管拔管 OR 拔管 | 188606 |
| 3 | 摘要=吞咽困难 OR 吞咽障碍 OR 口咽吞咽困难 OR 进食困难 OR 咽下困难 OR 拔管后吞咽困难 OR 获得性吞咽困难 OR 获得性吞咽障碍 | 27228 |
| 6 | 1 AND 2 AND 3 | 50 |

WanFang

Data searched: 12 March 2026

| **#** | **Search terms** | **Result retrieved** |
| --- | --- | --- |
| 1 | 主题:(儿童 OR 儿科学 OR 儿科 OR 患儿 OR 小儿 OR 青少年) | 2554113 |
| 2 | 主题:(气管插管 OR 人工气道 OR 人工通气 OR 机械通气 OR 气管拔管 OR 拔管) | 266683 |
| 3 | 主题:(吞咽困难 OR 吞咽障碍 OR 口咽吞咽困难 OR 进食困难 OR 咽下困难 OR 拔管后吞咽困难 OR 获得性吞咽困难 OR 获得性吞咽障碍) | 41579 |
| 4 | 1 AND 2 AND 3 | 97 |

Sinomed

Data searched: 12 March 2026

| **#** | **Search terms** | **Result retrieved** |
| --- | --- | --- |
| 1 | ( "儿童"[常用字段:智能] OR "儿科学"[常用字段:智能] OR "儿科"[常用字段:智能] OR "患儿"[常用字段:智能] OR "小儿"[常用字段:智能] OR "青少年"[常用字段:智能]) | 822452 |
| 2 | ( "气管插管"[常用字段:智能] OR "人工气道"[常用字段:智能] OR "人工通气"[常用字段:智能] OR "机械通气"[常用字段:智能] OR "气管拔管"[常用字段:智能] OR "拔管"[常用字段:智能]) | 155741 |
| 3 | ( "吞咽困难"[常用字段:智能] OR "吞咽障碍"[常用字段:智能] OR "口咽吞咽困难"[常用字段:智能] OR "进食困难"[常用字段:智能] OR "咽下困难"[常用字段:智能] OR "拔管后吞咽困难"[常用字段:智能] OR "获得性吞咽困难"[常用字段:智能] OR "获得性吞咽障碍"[常用字段:智能]) | 37849 |
| 4 | 1 AND 2 AND 3 | 58 |
